# Comparative Evaluation of Secondary Metabolite Chemodiversity of *Citrus* Genebank Collection in Greece: Can the Peel be More than Waste?

**DOI:** 10.1021/acs.jafc.4c00486

**Published:** 2024-04-13

**Authors:** Eftychia Martinidou, Michail Michailidis, Vasileios Ziogas, Domenico Masuero, Andrea Angeli, Theodoros Moysiadis, Stefan Martens, Ioannis Ganopoulos, Athanassios Molassiotis, Eirini Sarrou

**Affiliations:** † Institute of Plant Breeding and Genetic Resources, ELGO−DIMITRA, Thessaloniki 57001, Greece; ‡ Laboratory of Pomology, Department of Horticulture, Aristotle University of Thessaloniki, Thessaloniki-Thermi 57001, Greece; § Intsitute of Olive Tree, Subtropical Plants and Viticulture, ELGO−DIMITRA, Chania 73134, Greece; ∥ Fondazione Edmund Mach, Centro Ricerca e Innovazione, 38098 San Michele all’Adige, Trento, Italy; ⊥ Department of Computer Science, School of Sciences and Engineering, University of Nicosia, Nicosia 2417, Cyprus

**Keywords:** orange, mandarin, lemon, diversity, essential oil, flavonoids, carotenoids

## Abstract

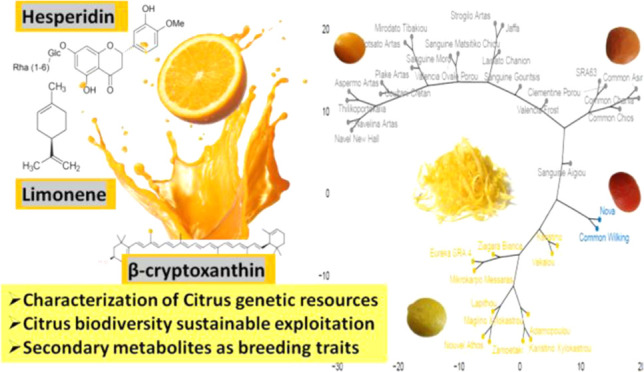

*Citrus* fruits are among the most economically important crops in the world. In the global market, the Citrus peel is often considered a byproduct but substitutes an important phenotypic characteristic of the fruit and a valuable source of essential oils, flavonoids, carotenoids, and phenolic acids with variable concentrations. The Mediterranean basin is a particularly dense area of autochthonous genotypes of *Citrus* that are known for being a source of healthy foods, which can be repertoires of valuable genes for molecular breeding with the focus on plant resistance and quality improvement. The scope of this study was to characterize and compare the main phenotypic parameters (*i.e.*, peel thickness, fruit volume, and area) and levels of bioactive compounds in the peel of fruits from the local germplasm of *Citrus* in Greece, to assess their chemodiversity regarding their polyphenolic, volatile, and carotenoid profiles. A targeted liquid chromatographic approach revealed hesperidin, tangeretin, narirutin, eriocitrin, and quercetin glycosides as the major polyphenolic compounds identified in orange, lemon, and mandarin peels. The content of tangeretin and narirutin followed the tendency mandarin > orange > lemon. Eriocitrin was a predominant metabolite of lemon peel, following its identification in lower amounts in mandarin and at least in the orange peel. For these citrus-specific metabolites, high intra- but also interspecies chemodiversity was monitored. Significant diversity was found in the essential oil content, which varied between 1.2 and 3% in orange, 0.2 and 1.4% in mandarin, and 0.9 and 1.9% in lemon peel. Limonene was the predominant compound in all *Citrus* species peel essential oils, ranging between 88 and 93% among the orange, 64 and 93% in mandarin, and 55 and 63% in lemon cultivars. Carotenoid analysis revealed different compositions among the *Citrus* species and accessions studied, with β-cryptoxanthin being the most predominant metabolite. This large-scale metabolic investigation will enhance the knowledge of *Citrus* peel secondary metabolite chemodiversity supported by the ample availability of *Citrus* genetic resources to further expand their exploitation in future breeding programs and potential applications in the global functional food and pharmaceutical industries.

## Introduction

The genus *Citrus* (*Rutaceae*) consists of polycarpic, evergreen, flowering plants that grow in tropical and/or subtropical climates around the globe.^1^ The most economically and industrially important representatives of the genus are oranges (*C. x aurantium var. sinensis L.)*, tangerines (*C. x aurantium var. deliciosa ined.)*, lemons (*C. x limon var. limon (L.) Burm. f.)*, limes (*C. x aurantifolia var. aurantifolia)*, and grapefruits (*Citrus paradise*).^2^*Citrus* species are one of the most important sources of vitamin C intake for humans, while containing a multitude of bioactive secondary metabolites. Meanwhile, citrus species have an essential role in the world market, finding applications in the food, cosmetic, and pharmaceutical industries.^3^

According to the Food and Agricultural Organization of the United Nations (FAO), the annual production of citrus fruits fluctuated between 126 and 143 thousand tons globally during the past decade (2011–2019). Roughly one-third of those fruits are processed, resulting in a significant amount of residues, which involve mainly the peel tissue of the fruit.^4^ The citrus peel consists of two parts: the exocarp (or flavedo), which is the outer, glandular layer of the skin, rich in essential oil (EO), and the mesocarp (or albedo), which is the soft, white inner part, abundant in pectin and cellulose.^5^ In global markets, the peel is often considered a byproduct that receives less attention than the endocarp (juicy sac) even though it can be a valuable source of EO and many other bioactive components such as polyphenols and carotenoids.^6^ During the processing of citrus fruits into juices, 50–60% of the fresh weight is not further used, thus resulting in a waste of resources.^7^

*Citrus* peel represents an abundance of several classes of secondary metabolites, including flavonoids, limonoids, carotenoids, and EO.^8^ Particularly, flavanone and flavone *C*- and *O*-glycosides as well as polymethoxyflavones are the principal groups of flavonoids found in citrus peels.^9,10^ Flavanone glycoside derivatives present anticancer and anti-inflammatory activities,^11^ while among them, hesperidin is the most prevalent in oranges.^9^ Tangeretin and nobiletin are some of the most studied polymethoxyflavones in *Citrus*, also known for their anti-inflammatory and anticancer activities.^12^ However, hydroxylated polymethoxyflavones often display similar but stronger effects. Another important group of secondary metabolites in citrus peels is the carotenoids. Especially, *b*-carotene, which exhibits high pro-vitamin A activity, is often used as a nutraceutical against cardiovascular diseases, multiple sclerosis, and cancer, due to its excellent single oxygen and free radical scavenging properties.^13,14^ Finally, citrus EO consists of variable volatile bioactive compounds that are mainly concentrated in the oil glands of the exocarp.^5^ Among them, limonene and γ-terpinene are the most abundant hydrocarbon monoterpenes present in the flavedo, with a wide range of antimicrobial, antioxidant, and anticancer functions.^15^

Noticeably, the diverse genetic resources are the backbone of crop improvement programs, and for diverse fruit species such as citrus, their importance is non-negotiable. The autochthonous *Citrus* species can be repertoires of valuable genes for molecular breeding with the focus on plant resistance and quality improvement. Besides their health-promoting properties for humans, from a plant perspective the production of these secondary metabolites composes their arsenal to resist pathogens and insects and at the same time their means to “communicate” and interact with the environment in general. Therefore, it is important to determine the phytochemical diversity of *Citrus* species for the exploitation of their biodiversity and its conservation for sustainable development and bioprospecting. For this purpose, 36 indigenous *Citrus* cultivars from Greece were phytochemically investigated regarding their EO, polyphenolic, and carotenoid qualitative and quantitative fingerprints of the peel using state-of-the-art analytical chromatographic techniques. This in-depth metabolomic investigation will enhance the knowledge on *Citrus* peel chemodiversity to further expand its exploitation in future breeding programs and potential applications in the global functional food and pharmaceutical industries, taking into account the reduction of *Citrus* waste biomass under a sustainable bioeconomy.

## Materials and Methods

### Chemicals

Analytical-grade general laboratory supplies were purchased from Sigma-Aldrich and Fisher Scientific (Milan, Italy). Methanol, ethanol, acetonitrile, and pentane used prior to the chromatographic analysis were liquid chromatography–mass spectrometry (LC–MS) grade, while methyl-*tert*-butyl ether and all relative gases used in systems (He, N, *etc.*) had the highest purity for chromatography. The Arium purification system (Sartorius AG, Goettingen, Germany) was used to purify deionized water. Authentic standards of polyphenolic metabolites and carotenoids were obtained from TransMIT PlantMetaChem (Gießen, Germany) and Phytolab GmbH & Co. (Vestenbergsgreuth, Germany). PTFE membranes (0.22 μm) were purchased from Merck Millipore (Darmstadt, Germany).

### Plant Material and Sampling

All *Citrus* fruits were picked in 2021–2022 at the commercial harvest stage from an *ex situ* germplasm collection preserved at Chania, Crete, Southern Greece (Institute of Olive Tree, Subtropical Plants and Viticulture, ELGO–DIMITRA). The fruits were obtained from orange trees (*C. x aurantium* var. *sinensis* L., 17 cultivars), lemons (*C. x limon* var. *limon* (L.) Burm. f., 11 cultivars), mandarins/clementines (*C. x aurantium* var. *deliciosa* ined., four cultivars; *C. x aurantium* var. *clementina* ined., two cultivars; *C. clementina ×* (*C. paradisi*. *× C. reticulata*), Nova cultivar), bergamots (*C. x limon* var. *bergamia* ined., three cultivars), and limes (*C. x aurantifolia* var. *aurantifolia*, two cultivars), all indigenous to Greece as described in Michailidis et al.^16^ (Table S1). The orchard was set up of 25 years old trees that were all grafted onto a *Citrus aurantium* var. *aurantium* L. (sour orange) rootstock and planted in the same block with 4 × 6 m^2^ spacing between rows and along the row. The germplasm was grown under open field conditions following regular and optimum agricultural practices.

The fruit collection was performed manually from three individual trees per cultivar, combining fruit from the inner and outer parts of the canopy, which was separated into four quadrants. A total of 24 fruits per tree were harvested, and three representative biological replicates were developed by randomly combining the fruit of each tree. The fresh fruit were transferred to the laboratory for assessment of fruit quality traits and then for postharvest processing; after washing them with tap water, the exocarp (flavedo) was separated from the rest of the fruit and subjected to different extraction processes, to evaluate their secondary metabolite fingerprint in terms of EO content and their volatile composition, polyphenols, and carotenoids profile.

### Fruit Quality and Peel Physiological Attributes

The fruit volume, area, height–width ratio, and peel thickness (mm) were measured using a digital caliper (0.01 mm, RS PRO 150 mm, RS Components Sdn Bhd, Malaysia) in 16 fruit per cultivar. Volume (cm^3^) and area (cm^2^) were calculated based on the equations *V* (volume) = 4/3 × π × (height/2) × (width/2)^2^ and *A* (area) = 4 × π × [(height + width)/4]^2^. In addition, the exocarp color was quantified using a Minolta CR200 colorimeter (Minolta, Osaka, Japan) in terms of lightness (L*), redness (a*), and yellowness (b*). The values for chroma (C*), hue angle (*H*°), and citrus color Index (CCI) were determined using the following equations: C* = (a*^2^ + b*^2^)^0.5^; *H*° = arctan (b*/a*); and CCI = (a* × 1000)/(b* × L*).^17^ Arithmetic data are provided in Table S2.

### Essential Oil Isolation

About 50 g of fresh peel tissue was subjected for 3 h to hydrodistillation using a Clevenger-type apparatus according to the European Pharmacopoeia, as previously reported by Sarrou et al.^18^ The EO content of each cultivar was determined based on the fresh weight of peel tissue (mL/100 g), and three hydrodistillations per cultivar were performed. The obtained EO was dried over anhydrous sodium sulfate in dark glass vials and directly injected for gas chromatographic analysis.

### Determination of Citrus Essential Oil Composition through Gas Chromatography–Mass Spectrometry–Flame Ionization (GC/MS, GC/FID)

For the qualitative profiling, EO analysis was carried out using a Shimadzu 17A Ver. A three-gas chromatograph that was interfaced with a QP-5050A mass spectrometer and supported by GC/MS Solution ver. 1.21 software was used. The volatile compounds were separated on a capillary Agilent HP-5MS 30 m, 0.25 mm, 0.25 m column under the following conditions: injection temperature set at 260 °C, interface line at 300 °C, ion source at 200 °C, EI mode: 70 eV, scan range: 41–450 amu, and scan time at 0.50 s. The oven temperature program was set at 55 °C (hold time 1 min), 55–110 °C (rate 1.5 °C min^–1^), 110–150 °C (3 °C min^–1^), and 150–220 °C (8 °C min^–1^) with constant temperature at 220 °C for 10 min, carrier gas He, 54.8 kPa, and split ratio: 1:30. The relative content of each compound was calculated as percent of the total chromatographic area, and the results were expressed as means of three biological replicates.^18^ The compounds were identified by comparing their retention indices (RI) to those of *n*-alkanes (C7–C22), their literature data to the relevant compounds, and their spectra to those of the MS libraries (NIST 98, Willey, Fragrance).

To determine the quantitative composition, the EO was analyzed in a Shimadzu Nexis GC-2030 series gas chromatograph system with a flame ionization detector (FID) and a Shimadzu AOC-20i Auto injector, using a Crossbond MEGA-5MS column (30 m × 0.25 mm, film thickness 0.25 μm) coated with 95% methyl polysiloxane. The oven temperature program followed was the same as that in GC-MS analysis. The injector and detector temperatures were set at 260 and 280 °C, respectively. The injection volume was 1 μL, He was used as a carrier gas (1 mL min^–1^), and the split ratio was 1:30.

### Polyphenolic Extraction and Profiling of the Citrus Germplasm through Ultraperformance Liquid Chromatography–Tandem Mass Spectrometry (UPLC-MS/MS)

Separated fresh citrus peel samples were frozen at −20 °C, freeze-dried (Freeze-dryer α 1–2 LD plus, Christ, Osterode, Germany), and pulverized with a laboratory mill (Retzch, Haan, Germany). To extract the polyphenolic metabolites, about 100 mg of dried and pulverized peel samples was mixed with 4 mL of 80% methanol and vortexed briefly. The extraction proceeded for 20 min under an orbital shaker at 25 °C and 10 min under sonication, following 48 h of maceration at 4 °C in the dark, as previously reported by Multari et al.^19^ The extracts were centrifuged for 10 min at 1800*g* (4 °C) and filtered through a MILLEX 13–0.22 μm PTFE membrane filter into a dark glass vial for analysis. Three extractions were performed on the *per* citrus cultivar. The extracts were injected directly, and the data were expressed as the means of three biological replicates *per* citrus cultivar.

A Waters Acquity UPLC system (Milford, MA) was employed for targeted UPLC-MS/MS (MRM) analysis following the method previously described by Vrhovsek et al.^20^ Water and acetonitrile (both containing 0.1% formic acid) were used as mobile phases for the gradient, and a Waters Acquity HSS T3 column, 1.8 μm, 100 mm × 2.1 mm (kept at 40 °C), was used for the separation of the phenolic metabolites. Mass spectrometry detection was performed on a Waters Xevo TQMS instrument equipped with an electrospray in-spray (ESI) source. The parameters of the MS detector were as follows: 3.5 and −2.5 kV capillary voltage in positive and negative mode, respectively; source temperature at 150 °C; desolvation temperature at 500 °C; cone gas flow at 50 L h^–1^; and desolvation gas flow at 800 L h^–1^. All compounds were identified by comparing the retention time and spectral characteristics of the peaks with those of high-performance liquid chromatography (HPLC)-grade standards. Multiple reaction monitoring (MRM) was used for quantification based on the peak area of the samples as described by Multari et al.^19^ Calibration curves of external standards from all polyphenolic compounds were injected for quantitative analysis, and the results were expressed as milligrams of each compound identified at 100 g^–1^ dry peel tissue. Peak annotation and processing were carried out using the Mass Lynx Target Lynx application manager (Waters).

### Carotenoid Extraction and Profiling by Ultraperformance Liquid Chromatography (UPLC*-*MS*-*DAD)

The extraction of carotenoids was performed under dim light by mixing about 200 mg of freeze-dried and pulverized peel tissue with 5 mL of methanol/acetone/hexane (25:25:50%, v/v/v) containing 0.1% BHT (w/v) as previously described by Multari et al.^19^ The mixture was vortexed and mixed for 10 min under an orbital shaker. Following that, samples were placed into an ultrasonic bath kept at 10 °C and 59 kHz for 5 min. After centrifuging the mixtures for 10 min at 1800*g* and 4 °C, the organic layers were collected into 50 mL Falcon tubes and the tissue was re-extracted twice more. The organic extracts were combined and dried at 35 °C under decreased pressure. The dry residues were saponified using 4 mL of 15% KOH in MeOH (w/v) solution overnight at room temperature in a shaking incubator. After saponification, 4 mL of NaCl solution (9%, w/v) and 5 mL of hexane:diethyl ether (3:1, v/v) were added, and the mixtures were placed on an orbital shaker for 10 min at room temperature, vortexed, and centrifuged for 5 min at 1800*g* at 4 °C. This step was repeated twice, and the organic layers were mixed, washed three times with 5 mL of water, and dried under a stream of nitrogen. The crude extracts were stored in −80 °C until HPLC analysis, were reconstituted right before injection in 0.2 mL of methyl-*tert*-butyl ether:ethanol (1:2, v/v), and filtered through 0.22 μm PTFE membranes into dark glass vials.

Carotenoid profiling was carried out on a single quadrupole HPLC-MS instrument equipped with a diode array detector (DAD) and a single quadrupole mass spectrometer QDa Acquity coupled to an Acquity UPLC instrument (Waters, Milford, MA). The chromatographic separation of carotenoid compounds was performed on a BEH C18 polymer column, 1.7 μm 2.1 × 100 mm^2^, equipped with a guard column (Waters), maintained at 55 °C, and using acetonitrile/water (1:1, v/v) (solvent A) and isopropanol (solvent B), both containing 0.1% (v/v) formic acid and buffered with 10 mM ammonium formate, as previously described by Dumont et al.^21^

The gradient was as follows: beginning condition: mobile phase flow of 0.25 mL min^–1^, 65% solvent A reducing to 45.7% solvent A in 4 min; down to 36.1% solvent A in 0.5 min; run isocratically for 3.5 min; decreasing to 0% solvent A in 6 min; and run isocratically for 1.5 min. Before going back to the initial conditions, a cleaning program was carried out injecting 10 μL of a blank solution (MTBE: EtOH, 1:2, by volume) three consecutive times and using a gradient at 0.25 mL min^–1^ at 0.6 min with 100% B and then back to the initial conditions 65% A in 3 min. The total run (sample run + cleaning method) was achieved in 20.3 min. The autosampler was operated at 4 °C. For carotenoid detection, the DAD acquisition was set between 270–600 nm in steps of 1.2 nm. The mass spectrometer was equipped with an ESI source in positive ion mode with 15 V cone voltage. Mass acquisitions were performed in full scan mode (100–1200 uma) and SIR mode. For quantitative analysis, calibration curves of available authentic standards (*all-E*)-violaxanthin, lutein epoxide, antheraxanthin, (*all-E*)-lutein, (*all-E*)-zeaxanthin, β-cryptoxanthin, and (*all-E*)-β-carotene were injected in concentrations between 5 and 100 ppm.

### Statistical Analysis

The data from gas and liquid chromatographic analysis were initially employed on a two-way ANOVA (indicating the *Citrus* species and the cultivar as the two factors), with the statistical package SPSS 11, V17.0 (SPSS Inc. Chicago, Illinois). In addition, each dataset regarding the *Citrus* species was processed with on-way ANOVA. Tukey’s test was used for mean comparison within the post hoc analysis at *a* = 0.05 level of significance. Clustvis online software (http://biit.cs.ut.ee/clustvis/)^22^ was used to perform principal component analysis (PCA) to investigate the variation patterns in metabolite datasets and to develop clustering heatmaps to visualize the secondary metabolite patterns. For the phylogenic dendrograms, the distance matrix computation was performed based on scaled data and the Euclidean distance. In the hierarchical cluster analysis, the “complete” agglomeration method was employed. The R package “factoextra” was used to visualize the results. To develop the PCA biplots, which overlay the score plot and the loading plot in each case, the R package “factoextra” was used, based on the standard “prcomp” R function’s output on scaled data. The latter analyses were performed with R programming language v.4.2.1.

## Results

### Phenotypic Differences and Variations in the Volatiles, Polyphenols, and Carotenoids among Different Species of the Greek *Citrus* Genebank

**Figure 1:**
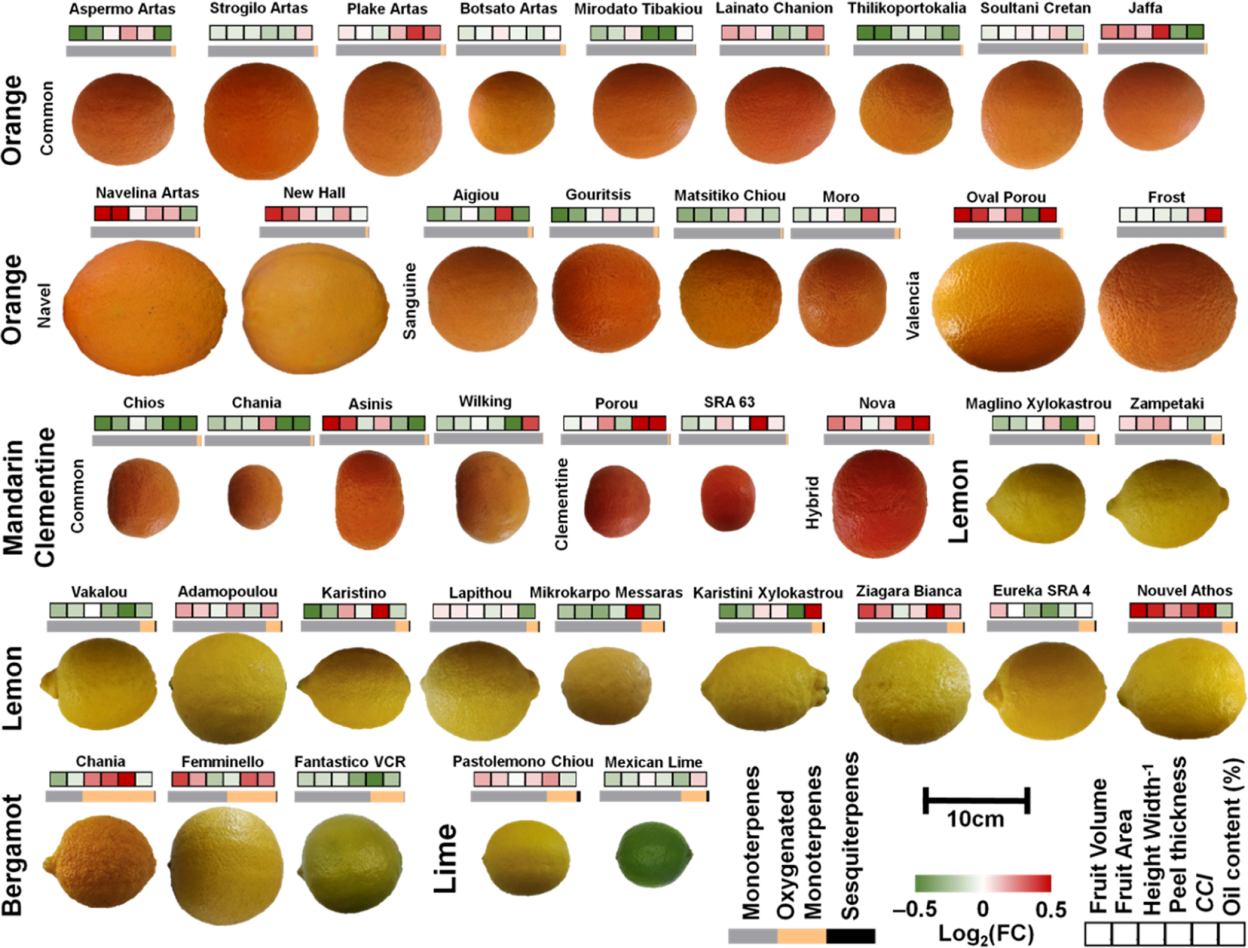
Phenotypic differences of fruits from orange, mandarin, lemon bergamot, and lime cultivars of *Greek* citrus genebank collection concerning the fruit volume and area, height width-1, peel thickness, CCI, and % essential oil content (upper heatmap boxes) and composition in monoterpene hydrocarbons, oxygenated monoterpenes, and sesquiterpenes (lower gray to black bar).

The phenotypic evaluation of the fruits from the different *Citrus* species revealed significant differences among the cultivars tested, concerning the fruit size (volume and peel area), color, and peel thickness (Figure 1 and Table S2). The fruit volume varied significantly between 132.7 and 383.6 cm^3^ in orange, 64 and 133.8 cm^3^ in mandarin, and 106.8 and 220.7 cm^3^ in lemon cultivars. Regarding the peel thickness, almost twofold thicker peel was observed in Jaffa fruit (6.4 mm) from “Mirodato Timbakiou” orange fruit and in “Nouvel Athos” lemon fruit (7.3 mm) compared with Eureka SRA 4.

**Figure 2:**
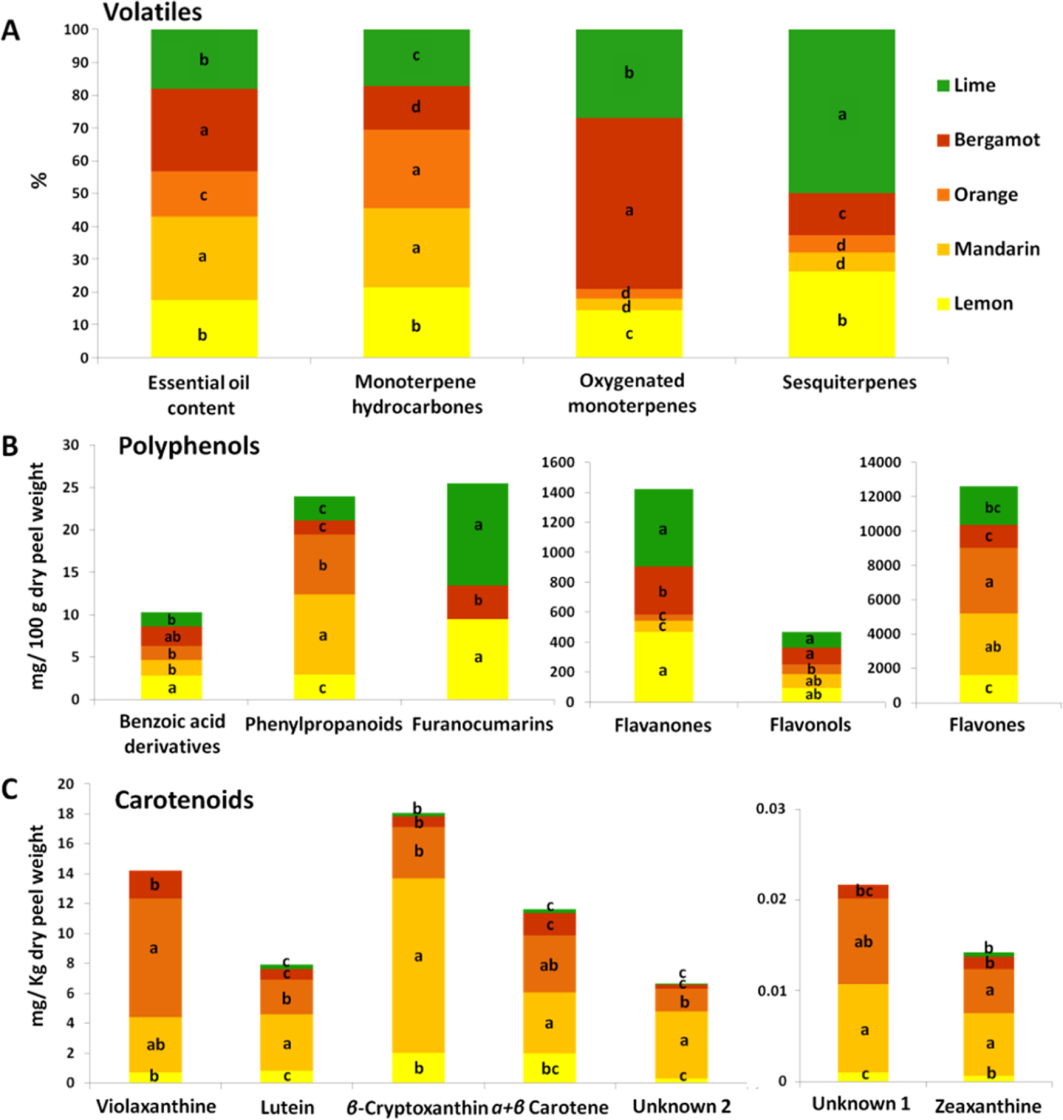
Variation of volatiles (A), polyphenols (B), and carotenoids (C) among different species of the Greek *Citrus* genebank collection. The bars represent the mean values of three independent biological replicates. Letters on the bars represent significant differences according to Tukey’s test for *p* ≤ 0.05.

To explore the aromatic profile of *Citrus* peel deriving from different species, including orange, lemon, mandarin, bergamot, and lime, we determined and compared the EO content and profile of different cultivars using GC-MS and GC-FID analysis. Regarding the EO content, which ranged from 1 to 1.8%, no notable disparities were observed across the species investigated (Figure 2A). Mandarin and bergamot showed the highest content of EO (1.8%), while orange had the lowest content (1%). A total of approximately 39 constituents, encompassing hydrocarbons, alcohols, and oxygenated compounds, were isolated from the *Citrus* species in varying levels, representing about 99.9% of the total EO composition. It was highlighted that the prevailing presence of certain volatile classes for each species, for example, monoterpene hydrocarbons, comprises a significant proportion of the total EO content for mandarin (95.24%) and orange (94.47%). Lemon and lime displayed noteworthy quantities of oxygenated monoterpenes, 12.91 and 24.13%, respectively, while bergamot represented a nearly equal distribution of both classes, with oxygenated monoterpenes comprising 46.75% and hydrocarbons accounting for 53.05% of the total EO content. Nevertheless, among the *Citrus* species, lime (2.88%) and lemon (1.52%) exhibited the highest concentrations of sesquiterpenes (Figure 2A).

Apart from the presence of volatile secondary metabolites, *Citrus* species are abundant in a diverse array of polyphenolic compounds, with the most predominant class being the flavonoids comprising flavanones, flavones, and flavanols. A total of 24 polyphenolic compounds, grouped in benzoic acid derivatives, phenylpropanoids, furanocoumarins, flavanones, flavonols, and flavones, were determined in varying concentrations across the different citrus species. Approximately equivalent amounts of benzoic acid derivatives were determined in all species, whereas the highest values of phenylpropanoids were detected in mandarin and orange. Furanocoumarins were identified exclusively in lime, lemon, and bergamot, with lime representing the highest amounts (12.06 mg, 100 g^–1^). The highest accumulated class was flavones, ranging from 3850.25 mg 100 g^–1^ of dried peel tissue in orange to 1316 mg 100 g^–1^ in bergamot (Figure 2B). Lime and lemon exhibited the most substantial quantities of flavanones, with concentrations of 517 and 469.81 mg 100 g^–1^, respectively, while orange displayed the most modest values at 40.64 mg 100 g^–1^. Flavonols exhibited a significantly higher concentration in bergamot and lime (113.75 and 106.03 mg 100 g^–1^, respectively) to 63.17 mg 100 g^–1^ in orange.

The predominant carotenoids identified in *Citrus* species fall within the subclass of xanthophylls, which are distinguished by their oxygenated chemical structure. During our comprehensive investigation across various species, we successfully isolated seven distinct xanthophylls, including violaxanthin, lutein, β-cryptoxanthin, α- + β-carotene (as a sum), and zeaxanthin, along with two unidentified substances (Figure 2C). Notably, β*-*cryptoxanthin emerged as the most prevalent, exhibiting a concentration of 11.69 mg kg^–1^ of dry peel tissue in mandarin. Furthermore, mandarin was observed to be the primary source of most of the identified xanthophylls, except violaxanthin, which was significantly more accumulated in the orange species (7.94 mg kg^–1^). Violaxanthin was the prominent carotenoid for bergamot with a mean value of 1.87 mg kg^–1^, closely followed by α- + β- carotene at 1.50 mg kg^–1^. In lemon, both violaxanthin and α- + β-carotene demonstrated the highest concentration values, with levels being closely comparable. Conversely, lime exhibited negligible amounts of carotenoids (Figure 2C). Overall, β-cryptoxanthin varied from 11.69 mg kg^–1^ in mandarin to 0.2 mg kg^–1^ in lime, and violaxanthin was accumulated highly in orange (7.94 mg kg^–1^), while the lowest concentration was observed in lemon (0.71 mg kg^–1^). Lutein demonstrated a notable variance, ranging from 3.79 mg kg^–1^ in mandarin to 0.28 mg kg^–1^ in lime. Similar concentration patterns were also evident for α- + β-carotene and unknown substance 2, while only trace amounts of zeaxanthin and unknown compound 1 were detected.

### Variation of the Essential Oil Composition among Different Cultivars of Lemon, Mandarin, and Orange

To gain a more comprehensive insight into the diversity of the peel’s EO composition, a comprehensive analysis was performed focused on the species with a higher number of representative cultivars, involving 11 lemon, 7 mandarin, and 17 orange cultivars from the Greek *Citrus* genebank. The total EO content was found to vary within the lemon species between 0.97 and 1.87%, with “Karistini-Xylokastrou” accumulating a twofold higher EO content in the peel compared with the “Lapithou” (Table S3). Within the lemon germplasm collection, 39 volatile compounds were isolated (Table S3). The predominant metabolites characterizing lemon EO comprise the monoterpene hydrocarbons limonene, β-pinene, and γ-terpene, alongside the oxygenated monoterpenes citral and neral. The most abundant compound, limonene, exhibited a range in concentration, varying significantly between 56.01% (“Mikrokarpo Messaras”) and 61.05% (“Karistino”). Furthermore, β-pinene was recorded to be higher in “Lapithou” among the Greek lemon cultivars. Additionally, nearly equivalent amounts of *γ-*terpene were detected for all the lemon cultivars, with “Maglino-Xylokastrou” exhibiting the highest concentration (10.84%), while “Mikrokarpo-Messaras” exhibiting the highest values of citral (7.76%) and neral (5.62%).

Regarding the peel EO composition of mandarin cultivars, 39 volatile metabolites were determined, accounting for more than 99.42% of the total identified oil. Among them, the monoterpene hydrocarbons limonene, γ-terpene, and myrcene, alongside the oxygenated monoterpenes linalool and α-terpineol were the most predominant (Table S4). Limonene accounted for more than 90% of the total EO content of most of the evaluated cultivars except “Common Asimis” and “Common Chios”, which represented 66.13 and 64.59%, respectively. Noticeably, the essential composition of these two cultivars varied from all the rest, accumulating substantial amounts of γ-terpene (17%), a twofold higher content in β*-*pinene and significantly higher content in α-thujene, β-pinene, cumol, and α-terpineol among the remaining mandarin cultivars. At least, “Clementine Porou” was found to accumulate the highest concentration of EO (2.1%) among all the cultivars examined, while “Common Chios” and “Asinis” expressed the lowest EO content (0.3%).

The peel EO isolated from the 17 orange cultivars was abundant in limonene, with relatively lower amounts of myrcene, octanal, and linalool (Table S5). “Mirodato Timpakiou” and “Sanguine Matsitiko Chiou” represented significantly higher values of limonene (≥93.80%) among the 17 cultivars, while “Sangouine Gouroutsis” was characterized by the highest amounts of linalool almost 2- to 3-fold higher compared with most of the orange cultivars. In terms of the EO content, “Valencia Frost” displayed a significantly higher content (2.93%), closely followed by “Valencia Ovale Porou” (2.63%) and “Lainato Chanion” (2.10%) among all the remaining investigated cultivars.

**Figure 3:**
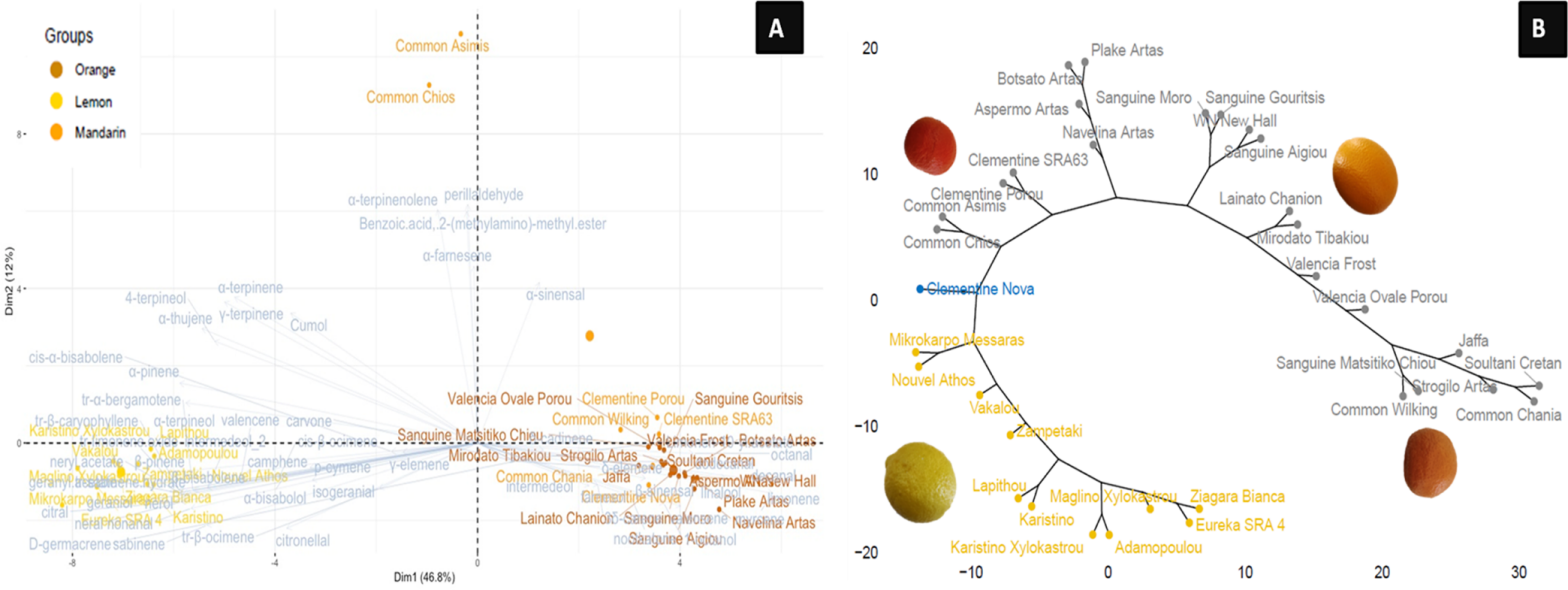
PCA biplot (A), with both PCA plot (samples) and plots of loadings (volatiles), and hierarchical clustering (B) of lemon, mandarin and orange *Citrus* cultivars based on their peel volatile compounds.

The biplot of PCA and hierarchical clustering analysis involving all cultivars investigated in this study revealed a clear grouping of the samples according to the species but also noticeable diversification, especially on mandarin cultivars indigenous to Greece (Figure 3A,B). Dimension 1 (PC1) separated the lemon cultivars clustered all together to the left part of the plot from mandarin and orange samples located in the right side. Dimension 2 (PC2) separated “Nova” mandarin from all the rest, while dimension 2 separated the two mandarin Common cultivars (“Chios” and “Asinis”). Overall the first two principal components explained 58.8% of the observed variance.

### Polyphenolic Fingerprints of the Different Lemon, Mandarin, and Orange Cultivars

**Figure 4:**
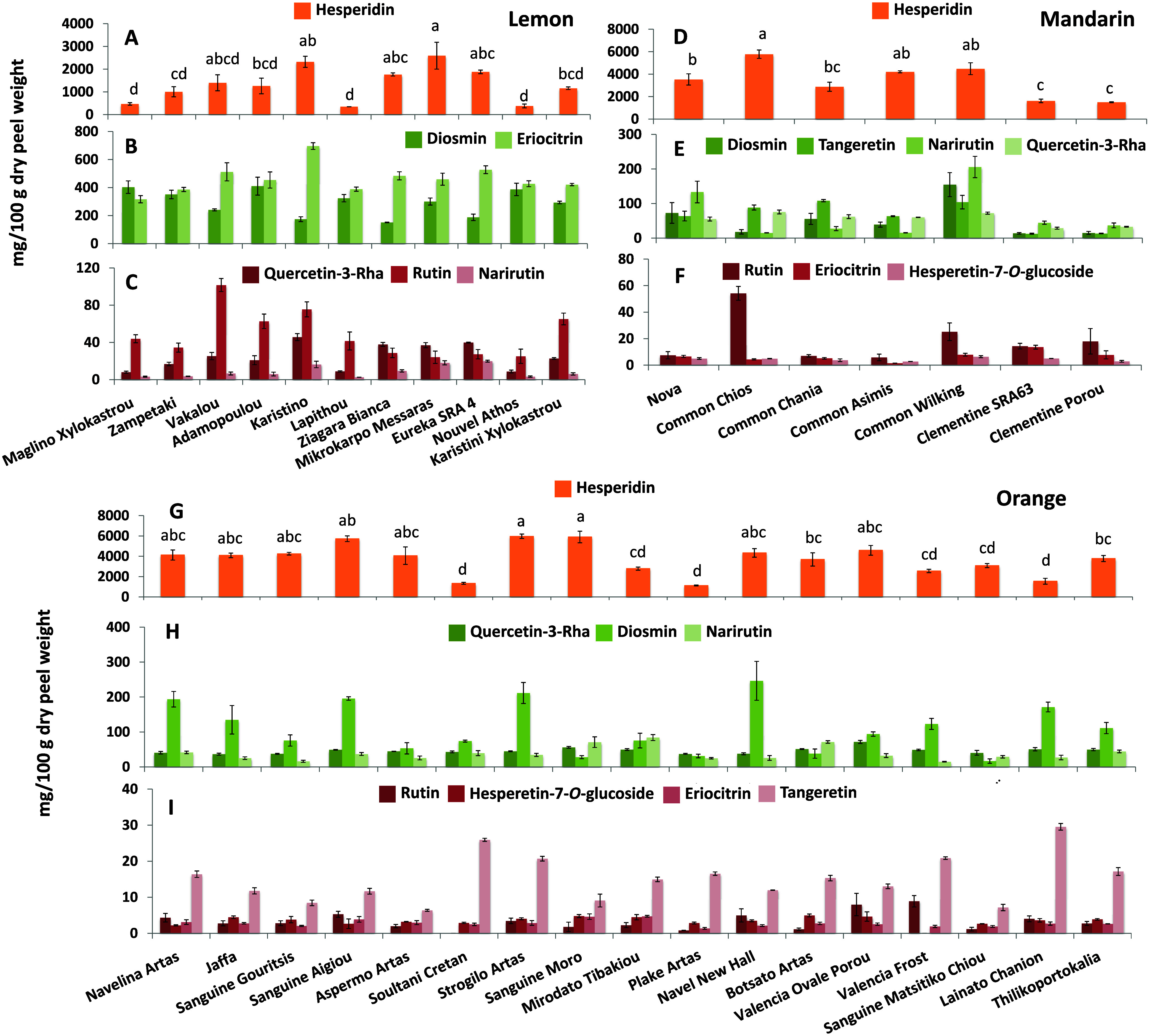
Variation of the most abundant flavonoids in the peel of 11 lemon (A–C) and seven mandarin (D–F) and 17 orange (G–I) cultivars from Greek *Citrus* genebank collection. The bars represent mean values of three independent biological replicates ± standard errors. Letters on the bars represent significant differences according to Tukey’s test for *p* ≤ 0.05.

To develop a detailed qualitative and quantitative polyphenolic fingerprint as well as to interpret the natural chemodiversity of all of the investigated Citrus cultivars, a targeted LC-MS/MS analysis was employed. A total of 18 distinct polyphenolic compounds were identified across the 11 lemon cultivars, with the most abundant metabolites being the flavanones hesperidin and eriocitrin, followed by the flavone diosmin (Figure 4A–C and Table S6). The major polyphenolic metabolite determined in lemon peel was hesperidin with concentrations ranging between 379.78 mg (in “Nouvel Athos”) and 2589.95 mg 100 g^–1^ (in “Mikrokarpo Messaras”). The eriocitrin content was about twofold higher in the peel of “Karistino” (696.07 mg 100 g^–1^), compared with “Maglino Xylokastrou” (316.84 mg 100 g^–1^). In addition, “Adamopoulou” represented the highest diosmin content, whereas the lowest was recorded in the “Ziagara Bianca” cultivar. Nevertheless, notable quantities of the flavonols rutin, quercetin-3-*O*-rhamnoside (Qu3rha), isorhamnetin-3*-O*-rutinoside (Isorha3rut), and furanocoumarin bergaptol were determined in varying amounts across the 11 lemon cultivars.

A total of 24 polyphenolic compounds were identified in the peels of mandarin cultivars. The most predominant metabolites identified were hesperidin, diosmin, tangeretin, narirutin, luteolin, and Qu3rha, while significant variations were observed among the investigated cultivars (Table S7). In particular, the peel from “Common Chios” contained a 2- to 3-fold higher content in hesperidin, compared with the remaining mandarin cultivars (Figure 4D). In lower concentration but still significant constituents of the polyphenolic profile, narirutin ranged significantly across the cultivars between 15.21 and 205.76 mg 100 g^–1^, same as tangeretin (12.84–108.33 mg 100 g^–1^) and diosmin (13.94–154.83 mg 100 g^–1^). The “Common Chios” mandarin accumulated significantly higher Qu3rha and rutin in the peel compared with the “Clemetines Porou” and ‘SRA64′ (Figure 4E), while eriocitrin and hesperetin-7-*O*-glc, an intermediate in the synthesis of the major flavonoid hesperidin, were detected only in traces (Figure 4F).

A similar pattern was observed among the 17 different cultivars of orange, with 18 polyphenolic compounds characterizing their profile (Table S8). Among them, hesperidin was again the most abundant flavanone ranging from 1121.05 mg 100 g^–1^ in “Plake Artas” to 5966.93 mg 100 g^–1^ in “Strogilo Artas”. Meanwhile, “Soultani Cretan”, “Plake Artas”, and “Lainato Chanion” were characterized by significantly lower amounts of hesperidin compared with all the remaining orange cultivars (Figure 4G). Diosmin was also present in significant amounts and showed high variability among the cultivars ranging from 16.74 mg 100 g^–1^ in “Sanguine Matsitiko Chiou” to 246.56 mg 100 g^–1^ in “Navel New Hall”. Qu3rha and narirutin were present in lower but diverse concentrations among the 17 orange cultivars, while rutin, hesperetin-7-*O*-gluc, tangeretin, and eriocitrin were detected only in traces (Figure 4H,I).

**Figure 5:**
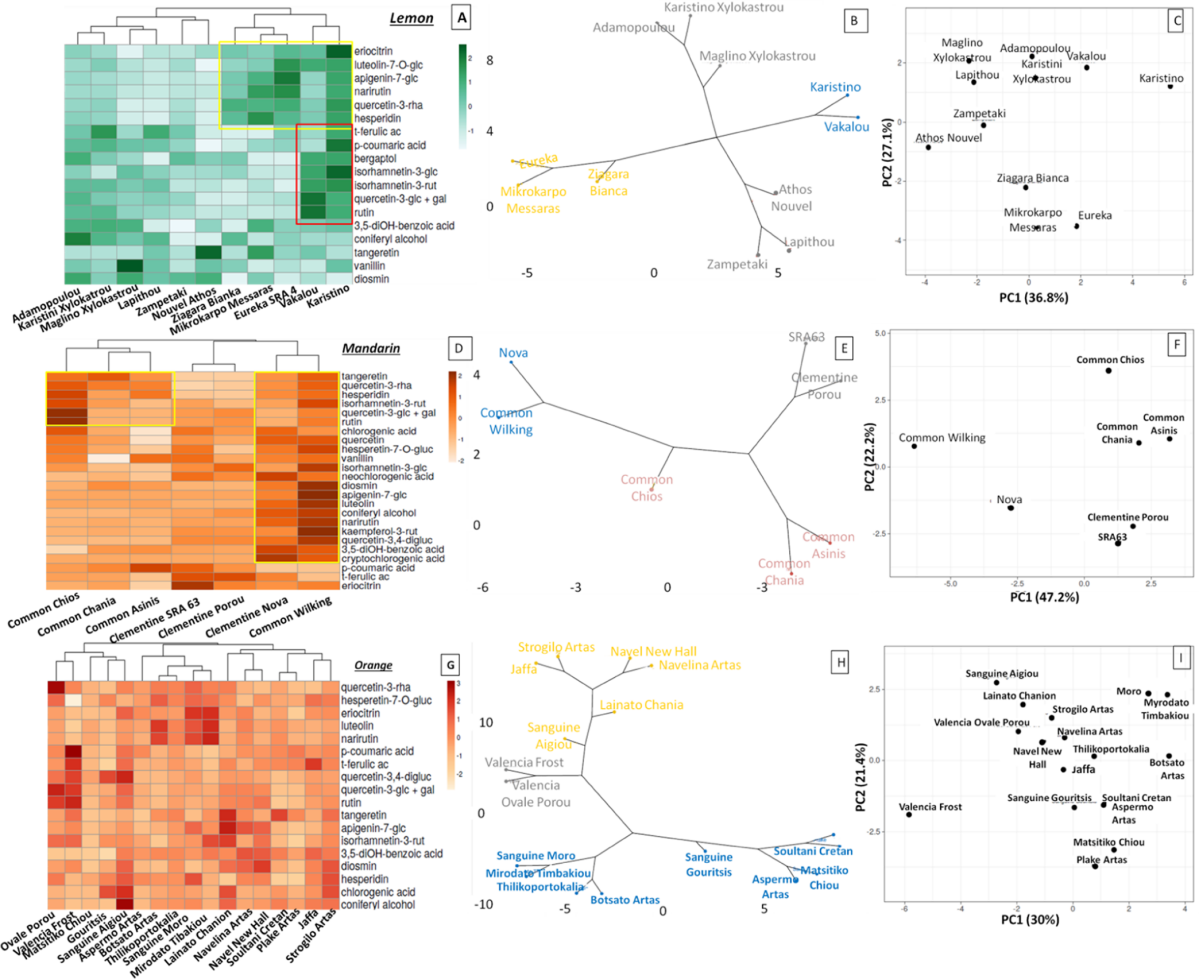
Clustered Heatmaps, hierarchical clustering, and principal component analysis of lemon (A–C), mandarin (D–F), and orange (G–I) cultivars based on their polyphenolic metabolites. The heatmaps visualize the log_10_-scaled metabolite concentration levels using different color scales from white to green in lemon, white to orange in mandarin, and white to red in orange cultivars.

Figure 5 depicts a detailed preview of the polyphenolic diversity observed in lemon, mandarin, and orange cultivars processed at the species level. According to the heatmap clustering, lemon cultivars are grouped in two wider clusters. This clustering seems to be mostly influenced by the higher levels of eriocitrin, narirutin, hesperidin, luteolin-7-*O*-glc, apigenin-7-*O*-glc, and que3rha in the peel of the cultivars “Eureka”, “Mikrokarpo Messaras”, “Ziagara Bianka”, “Karistino”, and “Vakalou”, while a subclustering was observed in the latter two samples mostly affected by the presence of quercetin and isorhamnetin derivatives, *p*-coumaric acid, and bergaptol (Figure 5A,B). Mandarin samples were grouped into three main clusters (Figure 5D,E) as well as orange cultivars (Figure 5G,H). In all three *Citrus* species, the PCA analysis confirmed this clustering, since more than 50% of the observed diversity was explained by the first two principal components in each case (Figure 5C,F,I).

**Figure 6:**
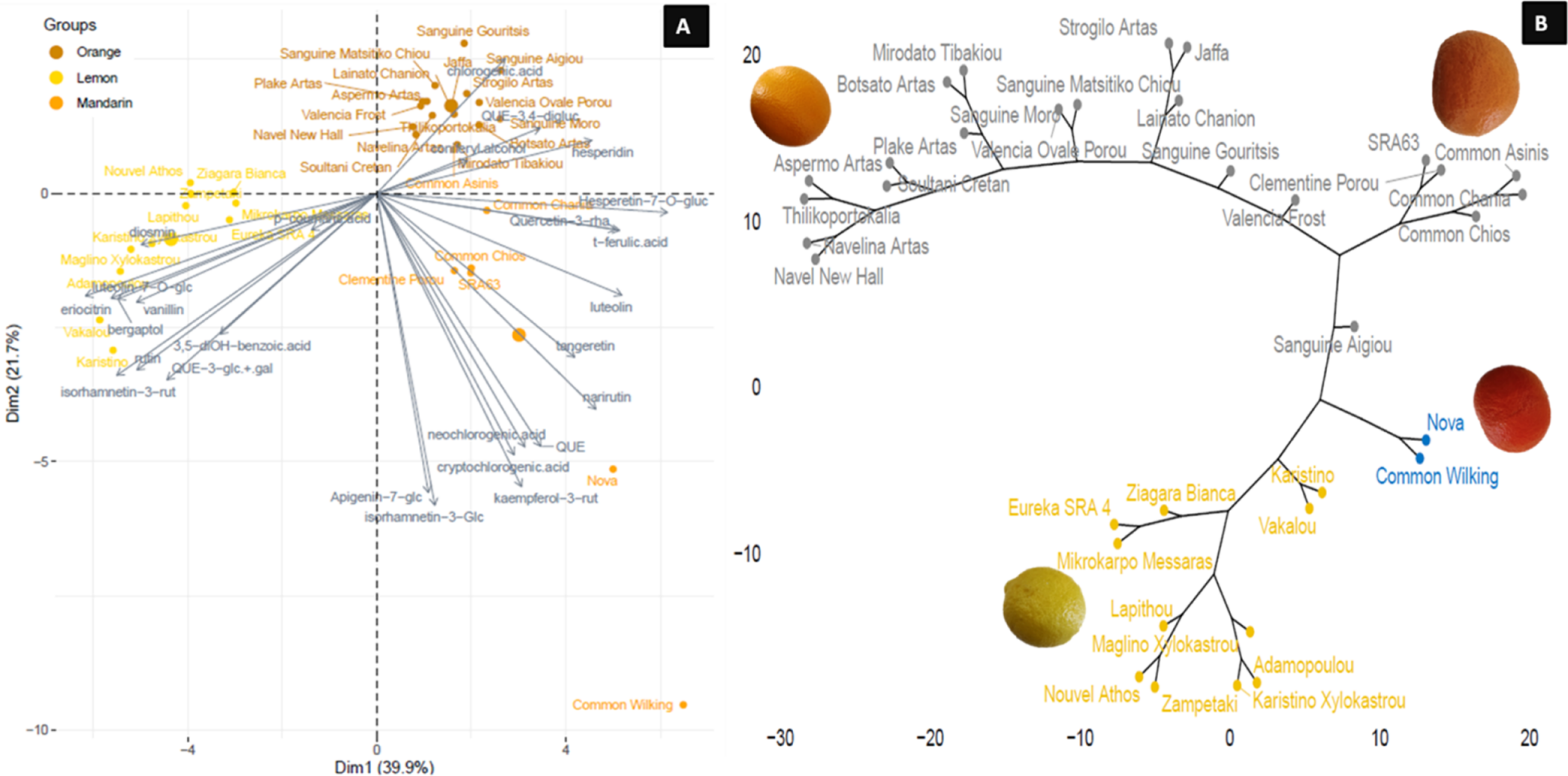
PCA biplot (A), with both PCA plot (samples) and plots of loadings (polyphenols), and hierarchical clustering (B) of lemon, mandarin, and orange *Citrus* cultivars based on their targeted polyphenolic profile.

Within the biplot of PCA based on the targeted polyphenolic profile of the samples, 61.6% of the observed diversification represented by the *Citrus* cultivars was explained by the first two principal components (Figure 6A). Similar to the PCA biplot based on volatiles, dimension 2 separated the lemon cultivars grouping to the left part of the plot, while mandarin and orange samples were spread to the right part of the plot. Dimension 1 separated orange samples located above the axes from mandarins, which were wider and spread below the axes. The “Nova” hybrid separated from all the rest, while dimension 1 split the two mandarin cultivars including in Common (“Chios” and “Asinis”). Noticeably, “Nova” and “Common Wilking” substitute a diverse cluster from the rest of mandarin samples.

**Figure 7:**
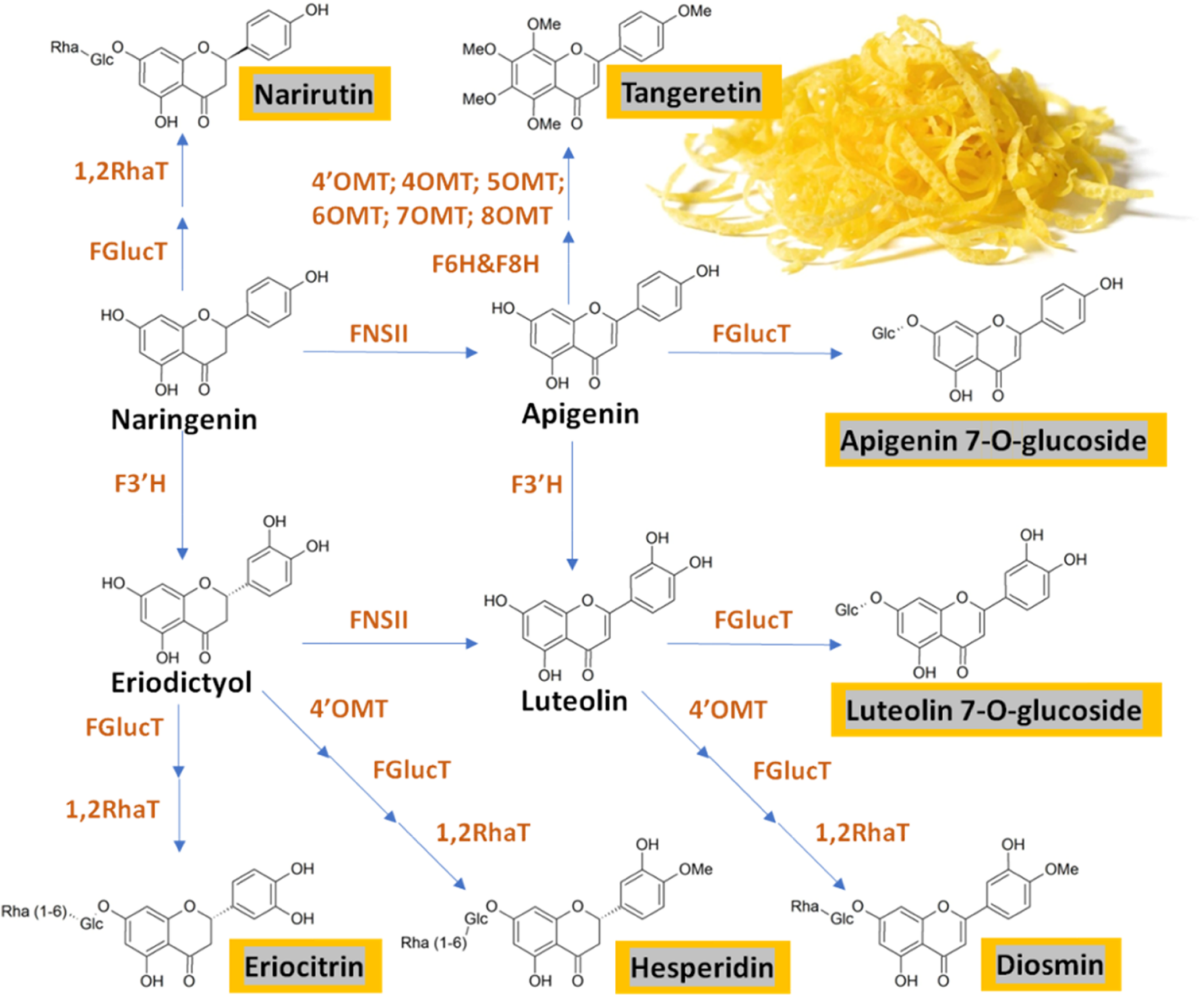
Simplified biosynthetic pathway of main *Citrus* flavonoids based on the detected compounds from targeted LC–MS/MS analysis, including the corresponding enzymes involved in each catalytic reaction step. F3′H—flavonoid 3′-hydroxylase, FNS II—flavone synthase II, 4′OMT—4′-*O*-methyltransferase; 4OMT—4-*O*-methyltransferase; 5OMT—5-*O*-methyltransferasȩ 6OMT—6-O-methyltransferasȩ 6OMT—6-*O*-methyltransferase; 7OMT—7-*O*-methyltransferase; 8OMT—8-*O*-methyltransferase; F6H—flavone 6-hydroxylase; F8H—flavone 8-hydroxylase; FGlucT—flavonoid glucosyltransferase; and 1,2RhaT—1,2-rhamnosyltransferase. Detected metabolites are given in orange boxes.

Given the identified polyphenolic compounds in the *Citrus* samples evaluated in this study, a putative biosynthetic pathway was designed *in silico* and is presented in Figure 7. As mentioned above, the main flavonoids belong to the groups of flavanones and flavones varying in their degree of hydroxylation and methylation but also in their further decoration with different glycosides. One predominant hydroxylation pattern concerns position 3′ of the flavonoid B-ring defining the flux toward 3′,4′ dihydroxylated derivatives such as eriodictyol, luteolin, and quercetin. A second path of hydroxylation relates to positions 6 and 8 of the A-ring (*e.g.*, tangeretin). Another important modification is the methylation of hydroxy groups in different positions. Here, the flavonoids can be divided into monomethylated (*e.g.*, hesperetin or diosmin) and polymethoxylated (*e.g.*, tangeretin) derivatives. The final obvious modification is achieved by glycosylation, resulting in mainly conjugation with rutinose (disaccharide composed of glucose and rhamnose) of flavanones and flavones, but monoglucosyled forms were also detected. Rutinose is added by two sequential and coordinated glycosyltransferases, where in the first step, a glucose moiety is attached to the hydroxy group at position C7 by a classical flavonoid glucosyltransferase (FGlucT) followed by the addition of rhamnose by 1,2-rhamnosyltransferase (1,2RhaT). Overall, the flux to one or the other branches seems to be species-specific, while the overall concentration is varying significantly within the cultivars of the species.

### Carotenoid Profiles of Different Cultivars from Lemon, Mandarin, and Orange Peel

A detailed species-to-species investigation revealed notable variations in carotenoid profiles among distinct lemon, mandarin, and orange Greek cultivars. Five carotenoid metabolites referring to violaxanthin, zeaxanthin, lutein, β-cryptoxanthin, and α + β carotene (as sum) were qualitatively and quantitatively identified *via* HPLC-MS-PDA analysis, together with two unknown compounds (1 and 2) with variable concentrations across the different cultivars for each species (Tables S9–S11).

**Figure 8:**
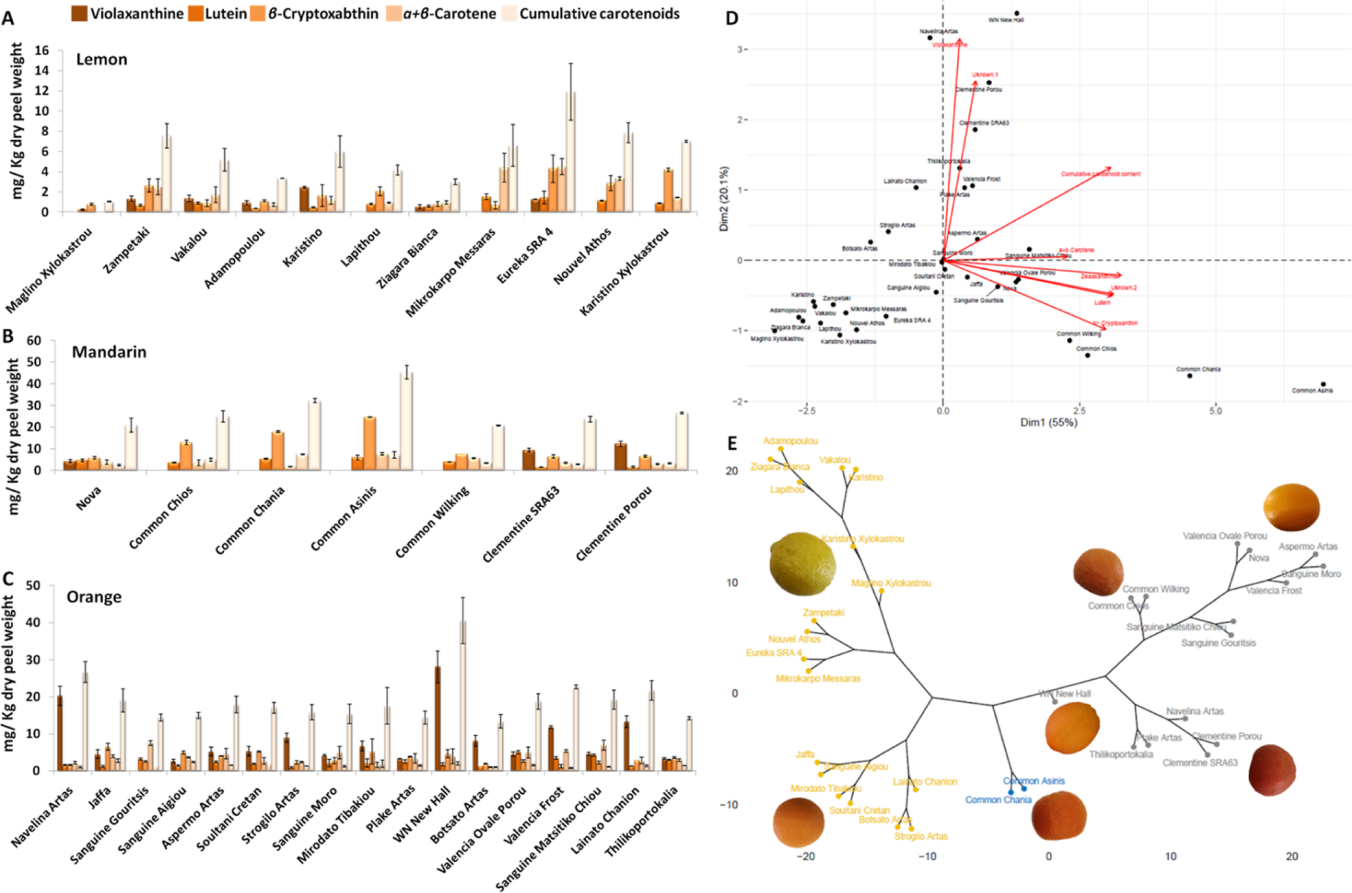
Variation of the carotenoid content (in individual compound and cumulative basis) in the peel of lemon (A), mandarin (B), and orange (C) cultivars from Greek *Citrus* genebank collection. Biplot analysis (D) and hierarchical clustering analysis (E) of the *Citrus* cultivars, based on their carotenoid profile. The bars represent mean values of three independent biological replicates ± standard errors.

Among the 11 different cultivars of lemon, β-cryptoxanthin was revealed to be one of the major carotenoid metabolites (0.77–4.301 mg kg^1–^) following α- + β-carotene (0–4.493 mg kg^1–^) and lutein (0.248–1.534 mg kg^1–^) (Table S9). The “Eureka SRA 4” exhibited a significantly higher cumulative carotenoid content due to the high abundance of β-cryptoxanthin and α- + β-carotene, from “Maglino Xylokastrou” (Figure 8A). On the other hand, the peel of “Karistino” fruit displayed the highest content of violaxanthin, while “Mikrokarpo Messaras” showcased the most substantial concentration of lutein.

Among the cultivars of mandarin, violaxanthin was only identified in the peel of the “Clementines Porou” and ‘SRA64′, while “Nova” varied significantly between the cultivars (Figure 8B and Table S10). β-Cryptoxanthin was the major carotenoid metabolite, with lutein, α- + β-carotene, and unknown compound 2 contributing significantly to the general carotenoid profile of mandarin cultivars. The peel of “Common Asinis” fruit revealed 2- to 4-fold higher levels in the cumulative carotenoid content, β-cryptoxanthin, α- + β-carotene, lutein, and the unknown compound 2 compared with the “Clementines Porou” and ‘SRA63′.

In contrast, among the 17 orange cultivars, violaxanthin was the most abundant metabolite, following β-cryptoxanthin, α- + β-carotene, lutein, and the unknown compound 2, with concentrations varying significantly among the cultivars examined (Figure 8C and Table S11). For instance, the peel samples of “WN New Hall” and “Navelina Artas” were superior in violaxanthin content compared with all the other cultivars. Remarkably, violaxanthin was not present at all in “Sanguine Gouritsis”, which demonstrated the highest accumulation of α- + β-carotene (7.549 mg kg^–1^). The peel of “Jaffa”, “Soultani Cretan”, and “Myrodato Timbakiou” was characterized by the presence of β-cryptoxanthin in concentrations ≥5 mg kg^–1^, whereas “Valencia Frost” exhibited the lowest (1.224 mg kg^–1^). Lutein production ranged from 0.889 mg kg^–1^ in “Strogillo Artas” to 5.059 mg kg^–1^ in “Valencia Ovale Porou”.

The biplot of the PCA depicts both samples and compounds representing the behavior of the carotenoid metabolic profile between the different intra- and interspecies *Citrus* cultivars and explained about 75% of the observed variations within the first two principal component (Figure 8D). In fact, dimension 2 separated the lemon samples located on the left from mandarin samples spread on the right. Dimension 1 separated the mandarin common cultivars (“Wilking”, “Chios”, “Chania”, “Asinis”) located at the right-hand bottom of the plot from the “Clementine” located on the upper right side of the PCA. The corresponding hierarchical clustering is displayed in Figure 8E.

## Discussion

The volatile constituents collectively impart diverse olfactory characteristics discerned in various *Citrus* cultivars. Noticeably, the precise composition and concentrations of these substances exhibit variability both across distinct *Citrus* species and within different cultivars of the same species. *Citrus* peel is the most productive tissue compared with the EO of leaves (petitgrain) and flowers (neroli),^18^ while the entire EO fraction is composed of up to 99% volatile and semivolatile compounds.^23^ Besides being widely exploited in industrial products, the *Citrus* EO (leaf and peel) volatome has been used for taxonomic studies and/or hybrid discriminations.^24,25^

The qualitative and quantitative GC analysis of the *Citrus* cultivars indigenous to Greece revealed the existence of diverse chemotypes in the mandarin germplasm collection. Orange and mandarin peel EO composition was characterized by the high presence of limonene (>90%) and myrcene (>2%), except for two cultivars (Common “Chios” and “Wilking”) that represented noticeably lower limonene content (up to 66%) and γ-terpinene (about 17%). Such diverse mandarin chemotypes have also been reported in fruits cultivated in other Mediterranean regions^26^ (cvs “Avana” and “Tardivo di Ciaculli”) and other mandarin cultivars.^27,28^ On the contrary, interspecific variation was observed concerning the composition of lemon EO, in which limonene (up to 62%), β-pinene and γ-terpinene (up to 10%), citral (up to 7.8%), and neral (up to 5.6%) composed the main volatile blend. The obtained data are largely in agreement with previous studies on sweet oranges’ and lemons’ qualitative EO composition.^29,30^ However, quantitative differences in individual volatiles may be attributed to genetic, cultivation techniques, ripening stage, and environmental factors as previously suggested.^24,30,31^

Apart from their role *in planta* and being involved in a majority of processes such as the defense system, reproduction, and hormone signaling, polyphenols exert an important role in food quality by improving their sensorial traits (sweet, bitter, color) and contributing to the prevention of degenerative diseases through their biological functions.^32^*Citrus* inter- and intraspecies polyphenolic profiles have been the interest of previous studies using targeted metabolite profiling.^10,33^ These studies documented noticeable flavonoid diversity and have been used as phylogenic metabolic markers that are influenced by plant-specific factors and analytical parameters, such as the extraction process and data analysis tools.^19,34^

The most predominant *Citrus* polyphenolic metabolites belong to flavanones (mainly di- and tri-*O*-glycosides), flavone glycosides (mainly di- and tri-*O*-glycosides and *C*-glycosides), and polymethoxyflavones^35,36^ and contribute to the quality of both fresh and processed industrial products. For instance, hesperidin (one of the major *Citrus* flavanone glycosides) has been reported as a potential cloudifier in citrus juices,^37,38^ while polymethoxylated flavones, which are centrally localized to *Citrus* peel oil glands, exhibit anticarcinogenic, antitumor, and potential neuroprotective activities.^39,40^

In the present study, significant inter- and intraspecific polyphenolic diversity of flavedo samples was observed across 36 lemon, mandarin, and orange Greek-originated cultivars, affecting their different grouping according to the hierarchical clustering analysis. In general, the concentration of the major individual flavonoid metabolites identified in the present study is comparable to reports on similar *Citrus* species^41,42^ following relatively comparable extraction solvents and chromatographic analysis. The peel flavonoid composition of lemon cultivars from the Greek gene bank investigated herein consisted mostly of the flavanones hesperidin and eriocitrin and the flavone diosmin (Figure 3A–C). All three compounds are 7-*O-*rutinosides containing glucose and rhamnose moieties, indicating strong activity of the two involved enzymes, flavonoid 7-*O*-glucosyltransferase (FGlucT) and 1,2-rhamnosyltransferase (1,2RhaT), respectively. The latter is the key enzyme in the biosynthesis of the bitter flavonoids found in *Citrus* species.^43^ Thus, the above finding indicates a strong bias toward 3′-hydroxylase, high and selective flavonoid 3′-hydroxylase (F3′H) activity, and 4′-methylation (hesperidin and diosmin) (Figure 7). On the other hand, mandarin and orange peels contain relatively high amounts of hesperidin, diosmin, tangeretin, narirutin, luteolin, and Qu3rha according to the cultivar (Figures 4D–F and 5). The presence of monohydroxylated flavonoids (B-ring; narirutin and tangeretin) indicates a lower shift toward the dihydroxylated flavonoids found in lemon, which might be due to the lower activity of F3′H leaving substrate for the glycosylation of naringenin and hydroxylation/polymethylation of apigenin toward narirutin and tangeretin, respectively.

Carotenoids are the pigments contributing to the coloration of *Citrus* peel and pulp, while being a critical commercialization trait even for table fruits, providing the first perception for the consumer’s acceptance.^44^*Citrus* fruits (exocarp and endocarp tissues) are rich in pro-vitamin A carotenoids, *i.e.*, α- and β-carotene and β-cryptoxanthin, as well as xanthophylls like violaxanthin and antheraxanthin.^19,45^ The majority of *Citrus* xanthophylls are esterified with diverse fatty acids, while their pattern can be quite complex according to the genetic background and sample processing.^46^ Saponification has been often employed to simplify the determination of the *Citrus* carotenoid content, revealing violaxanthin as one of the most abundant xanthophylls in orange, mandarin, lemon peel, and pulp extracts. Despite many reports on *Citrus* EO and polyphenolic analysis, the information on the *Citrus* peel carotenoid content is relatively scarce. The comparison of the present data with already published data, therefore, was not easy due to different expressions of the concentration (*i.e.*, based on fresh weight or referring to mg L^–1^), different fruit tissues (pulp, juice), analytical techniques (HPLC-MS), fruit maturation stages, a.o; employing the same extraction procedure revealed comparable carotenoid content in Italian *Citrus* species relative to the present study.^19^ The Common mandarin cultivars (“Asimis”, “Chania”, “Chios”, and “Wilking”) displayed a strong presence of β-cryptoxanthin, whereas the “Clementine” and the hybrid “Nova” peel pigments exhibited a rich combination of β-cryptoxanthin and violaxanthin. Significant variations were also recorded in Greek orange cultivars, where violaxanthin was determined to be the main carotenoid in “Navelina Artas”, “WN New Hall”, “Valencia Frost”, and “Lainato Chania”, while the carotenoid content of all the rest was composed of lower amounts of all identified metabolites. The hypothesis that the genetic background plays a pivotal role in carotenoid biosynthesis in *Citrus* was also supported by other studies suggesting that violaxanthin, β-cryptoxanthin content, and their combinations were markers for the classification of different *Citrus* genotypes.^13,27^ Conversely to orange and mandarin, lemon carotenoids are colorless (*i.e.*, phytoene, phytofluene) and chloroplastic carotenoids such as lutein and α- and β-carotene may also be present.^13,47^ Similar to the findings of the present study, β-cryptoxanthin was reported to be an abundant carotenoid in the peel and/or pulp of other lemon cultivars (*i.e.*, “Meyer”, “Eureka”); however, the course of this phenomenon is still unknown and could be attributed to the parental genetic makeup through crossing with oranges or mandarins carrying β-cryptoxanthin biosynthetic genes.^48^ Overall, the fruit peel from the Greek *Citrus* germplasm collection represented highly diverse carotenoid inter- and intraspecific fingerprints, which reflected their classification in variable clusters. Lately, the evolution of functional genomics (transcriptomics combined with metabolomics) revealed that besides the uncertain genetic origin of main *Citrus* cultivated species and cultivars, mutational events may also be responsible for the diversification of the genotypes together with differentially expressed genes at a transcriptional level, the enzymatic mechanism (substrate specificity, balance expression between upstream and downstream biosynthetic genes), and their regulators (transcription factors).^47,49,50^

Apart from their role *in planta*, *Citrus* peel secondary metabolites are considered high-added-value products for humans and may be recovered from citric waste. Within the agroindustrial sector, the *Citrus* processing industry is particularly important. About 50–60% of all *Citrus* fruits produced worldwide are orange fruits; however, other citrus species like grapefruit, lemon, mandarin, and lime are also significant to the *Citrus* industry.^51^ The output of *Citrus* fruits globally has increased significantly in recent years, reaching 98 million tons in 2020–2021 according to USDA 2020 forecasts. During *Citrus* processing (juicing and canning), eventually 120 million tons of industrial *Citrus* processing waste end up in the environment annually; hence, peel is an important source of sugars, polyphenols, pectin, carotenoids, EOs, and vitamin C as supported by the present and previous studies.^3^ In addition, *Citrus* EO is in great demand worldwide, accounting for about 500 billion $ in the international market due to its extended use in food, pharmaceutical, cosmetic, perfumery, and confectionery industries.^52^ Hence, the recovery of bioactive compounds, such as *Citrus* polyphenols, has been the topic of recent studies^53^ taking into account the increasing interest in dietary supplements, raw extracts in cosmetics, and natural additives in food products.^54^ For this reason, it is of great value to develop a database with the phytochemical potential of the *Citrus* germplasm/cultivars to meet industrial demand and criteria for these valuable plant “byproduct” quality.

The comprehensive evaluation of secondary metabolites in the *Citrus* peel from the Greek genebank collection presents a vivid picture of the chemodiversity inherent in these *Citrus* species and paves the way for future dissection of the biosynthesis of metabolic pathways in *Citrus*. Superior *Citrus* germplasm indigenous to Greece was identified in this study, which could be exploited in future breeding programs for qualitative fruit traits. The essential oil content was higher accumulated in the peel of Karystini Xylokastou (lemon), Clementine Porou (mandarin), and Valencia Frost and Ovale Porou (orange). With regard to the polyphenol content, Karistino and Mikrokarpo Messaras, Common Chios, and Strogilo Artas were the higher hesperidin producer cultivars, while Common Asinis, WN New Hall, and Navelina Artas had higher contents of cumulative carotenoids. Apart from the detailed metabolic screening, these data could be helpful in the selection of breeding parents for new metabolite-specific (aromatic, flavonoid-rich, and carotenoid-rich) germplasm. In addition and most importantly, this study aligns with and expands upon previous research, emphasizing the rich biochemical profile of *Citrus* peels and their potential applications in food and pharmaceutical industries, which contain significant levels of high-added value natural products with nutraceutical claims, while at the same time promoting the conservation of *Citrus* species biodiversity. This also corresponds to the growing interest in utilizing agricultural byproducts in a sustainable and economically viable manner, contributing to the broader goals of bioeconomy and biodiversity conservation.

## Supporting Information Available

Citrus cultivars of orange, mandarin, lemon, bergamot, and lime preserved in the Greek gene bank collection (Table S1); quantitative determination of Citrus exocarp physiological data (Table S2); composition of peel essential oil from 11 lemon cultivars of Greek Citrus Genebank (Table S3); composition of peel essential oil from seven mandarin cultivars of the Greek Citrus Genebank (Table S4); composition of peel essential oil from 17 orange cultivars of the Greek Citrus Genebank (Table S5); targeted polyphenolic profile of peel from 11 lemon cultivars of the Greek Citrus Genebank (Table S6); targeted polyphenolic profile of peel from seven mandarin cultivars of the Greek Citrus Genebank (Table S7); targeted polyphenolic profile of peel from 17 orange cultivars of the Greek Citrus Genebank (Table S8); carotenoid content of peel from 11 lemon cultivars of the Greek Citrus Genebank (Table S9); carotenoid content of peel from seven mandarin cultivars of the Greek Citrus Genebank (Table S10), and carotenoid content of peel from 17 orange cultivars of the Greek Citrus Genebank (Table S11) (XLSX)
